# Avian Influenza Viruses in Wild Birds: Virus Evolution in a Multihost Ecosystem

**DOI:** 10.1128/JVI.00433-18

**Published:** 2018-07-17

**Authors:** Divya Venkatesh, Marjolein J. Poen, Theo M. Bestebroer, Rachel D. Scheuer, Oanh Vuong, Mzia Chkhaidze, Anna Machablishvili, Jimsher Mamuchadze, Levan Ninua, Nadia B. Fedorova, Rebecca A. Halpin, Xudong Lin, Amy Ransier, Timothy B. Stockwell, David E. Wentworth, Divya Kriti, Jayeeta Dutta, Harm van Bakel, Anita Puranik, Marek J. Slomka, Steve Essen, Ian H. Brown, Ron A. M. Fouchier, Nicola S. Lewis

**Affiliations:** a Department of Zoology, University of Cambridge, Cambridge, United Kingdom; b Department of Viroscience, Erasmus MC, Rotterdam, Netherlands; c National Centre for Disease Control, Tbilisi, Georgia; d Institute of Ecology, Ilia State University, Tbilisi, Georgia; e J. Craig Venter Institute, Rockville, Maryland, USA; f Icahn School of Medicine at Mount Sinai, New York, New York, USA; g Animal and Plant Health Agency-Weybridge, Weybridge, United Kingdom; St. Jude Children's Research Hospital

**Keywords:** avian influenza, ecology, evolution, influenza, phylogenetics, viruses

## Abstract

Wild ducks and gulls are the major reservoirs for avian influenza A viruses (AIVs). The mechanisms that drive AIV evolution are complex at sites where various duck and gull species from multiple flyways breed, winter, or stage. The Republic of Georgia is located at the intersection of three migratory flyways: the Central Asian flyway, the East Africa/West Asia flyway, and the Black Sea/Mediterranean flyway. For six complete study years (2010 to 2016), we collected AIV samples from various duck and gull species that breed, migrate, and overwinter in Georgia. We found a substantial subtype diversity of viruses that varied in prevalence from year to year. Low-pathogenic AIV (LPAIV) subtypes included H1N1, H2N3, H2N5, H2N7, H3N8, H4N2, H6N2, H7N3, H7N7, H9N1, H9N3, H10N4, H10N7, H11N1, H13N2, H13N6, H13N8, and H16N3, and two highly pathogenic AIVs (HPAIVs) belonging to clade 2.3.4.4, H5N5 and H5N8, were found. Whole-genome phylogenetic trees showed significant host species lineage restriction for nearly all gene segments and significant differences in observed reassortment rates, as defined by quantification of phylogenetic incongruence, and in nucleotide sequence diversity for LPAIVs among different host species. Hemagglutinin clade 2.3.4.4 H5N8 viruses, which circulated in Eurasia during 2014 and 2015, did not reassort, but analysis after their subsequent dissemination during 2016 and 2017 revealed reassortment in all gene segments except NP and NS. Some virus lineages appeared to be unrelated to AIVs in wild bird populations in other regions, with maintenance of local AIVs in Georgia, whereas other lineages showed considerable genetic interrelationships with viruses circulating in other parts of Eurasia and Africa, despite relative undersampling in the area.

**IMPORTANCE** Waterbirds (e.g., gulls and ducks) are natural reservoirs of avian influenza viruses (AIVs) and have been shown to mediate the dispersal of AIVs at intercontinental scales during seasonal migration. The segmented genome of influenza viruses enables viral RNA from different lineages to mix or reassort when two viruses infect the same host. Such reassortant viruses have been identified in most major human influenza pandemics and several poultry outbreaks. Despite their importance, we have only recently begun to understand AIV evolution and reassortment in their natural host reservoirs. This comprehensive study illustrates AIV evolutionary dynamics within a multihost ecosystem at a stopover site where three major migratory flyways intersect. Our analysis of this ecosystem over a 6-year period provides a snapshot of how these viruses are linked to global AIV populations. Understanding the evolution of AIVs in the natural host is imperative to mitigating both the risk of incursion into domestic poultry and the potential risk to mammalian hosts, including humans.

## INTRODUCTION

Avian influenza viruses (AIVs) have been identified in a wide diversity of wild and domestic bird species, but wild waterbirds of the orders Anseriformes and Charadriformes, such as ducks, geese, swans, and shorebirds (1, 2), form their natural reservoir. These birds maintain a diverse group of low-pathogenic avian influenza A viruses (LPAIVs), which cause limited morbidity in these host species in experimental settings (3). The effect of AIV infection in wild birds in nonexperimental settings is more contradictory. Body mass was significantly lower in infected mallards (Anas platyrhynchos), and the amount of virus shed by infected juveniles was negatively correlated with body mass. However, there was no general effect of infection on staging time (duration of stopover for migratory birds), except for juveniles in September, and LPAIV infection did not affect the speed or distance of subsequent migration (4). Conversely, a recent mallard study demonstrated no obvious detriment to the bird, as movement patterns did not differ between LPAIV-infected and uninfected birds. Hence, LPAIV infection probably does not affect mallard movements during stopover, consequently resulting in the potential for virus spread along the migration route (5). The precise role of migrants and resident birds in amplifying and dispersing AIVs, however, remains unclear. In another study, the migrant arrivals rather than seeding of a novel variant into a resident population played a role in virus amplification (6). It has also been suggested that switching transmission dynamics might be a critical strategy for pathogens, such as influenza A viruses, associated with mobile hosts, such as wild waterbirds, and that both intraspecies transmission and interspecies transmission are important to maintaining gene flow across seasons (7).

AIVs continue to cause both morbidity and mortality in poultry worldwide. Increased mortality is strongly related to infection with highly pathogenic influenza A viruses (HPAIVs), characterized by mortality in gallinaceous poultry (8). Periodically, human infections associated with HPAIV of both the H5 and H7 subtypes have been detected. In particular, parts of Asia and Africa have been significantly affected by the Eurasian (goose/Guangdong/1996) lineage H5 HPAIV epizootic for 2 decades, which has become enzootic in some areas and which involves multiple waves of influenza with evolving viruses in others (9). More recently, H5Nx reassortants of the Eurasian lineage HPAIVs from clade 2.3.4.4 have been introduced into wild birds from poultry and spread to new geographic regions (10).

The Caucasus, at the border of Europe and Asia, is important for migration and overwintering of wild waterbirds. Three flyways, the Central Asian, East Africa/West Asia, and Black Sea/Mediterranean flyways, converge in this region (11, 12). Understanding the ecology and evolution of AIVs in wild birds is complex, particularly at sites where multiple species cohabit and in those ecosystems which support different annual life cycle stages and where multiple migratory flyways intersect.

At a population level, Eurasian dabbling ducks were found to be infected more frequently than other ducks and Anseriformes (13), with most AIV subtypes being detected in ducks; the exceptions were the H13 and H16 subtypes, which were detected primarily in gulls (13, 14). Temporal and spatial variation in influenza virus prevalence in wild birds was observed, with AIV prevalence varying by sampling location. In this study site in the Republic of Georgia, we observed a peak prevalence in large gulls during the autumn migration (5.3 to 9.8%) but a peak prevalence in black-headed gulls (Chroicocephalus ridibundus) in spring (4.2 to 13%) (15). In ducks, we observed an increased AIV prevalence during the autumn postmolt aggregations and migration stopover period (6.3%), but the levels were lower than those observed in other more northerly postmolt areas in Eurasia.

In North America, studies have primarily focused on Anseriformes species with sampling during the late summer and autumn southern migration (16–18), rather than longitudinally throughout the annual life cycle of the host or within an ecosystem. The southwestern Lake Erie Basin is an important stopover site for waterfowl during migration periods, and over the past 28 years, 8.72% of waterfowl sampled in this geographic location have been positive for AIV recovery during summer and autumn (June to December) (19). More recent studies which targeted overwintering and returning migratory birds from February to April showed the presence of diverse AIV subtypes in waterbirds at northern latitudes in the United States (19).

Previous genetic studies of the viruses isolated from wild birds have focused on virus gene flow involving multiple hosts at an intra- or intercontinental level, rather than on virus gene flow among species within an ecosystem (18, 20–22). Indeed, the conclusions of such studies have been somewhat limited at times by statistical power owing to insufficient sequence data from enough hosts relevant to virus dynamics across the geographic study area (23). In Eurasia, frequent reassortment and cocirculating lineages were observed for all eight genomic RNA segments over time. Although there was no apparent species-specific effect on the diversity of the AIVs, there was a spatial and temporal relationship between the Eurasian sequences and significant viral migration of AIVs from West Eurasia toward Central Eurasia (24).

This study presents novel findings concerning the ecology and evolution of both the LPAIVs and HPAIVs circulating in wild birds in a key active surveillance site in Eurasia.

We investigated the diffusion of AIV gene segments within different wild bird hosts occupying the same ecosystem. There was substantial diversity in surface glycoprotein hemagglutinin (HA) and neuraminidase (NA) subtypes, which varied from year to year and with the host species. M, NS, NP, PB1, PB2, and PA (henceforth referred to as “internal” gene segments) also showed host restriction to various degrees. There were differences in genetic diversity, reassortment rates, and interspecies transmission rates in the internal gene segments associated with different host species and HA subtypes. We also examined how closely related the Georgian AIV gene segments were to AIV gene segments globally. We found evidence for a genetic interrelationship of Georgian AIVs with AIVs in mainly Africa and Eurasia, but several lineages appeared to be maintained locally.

(This article was submitted to an online preprint archive [25].)

## RESULTS

### Prevalence, subtype diversity, and host specificity of AIVs.

**FIG 1:**
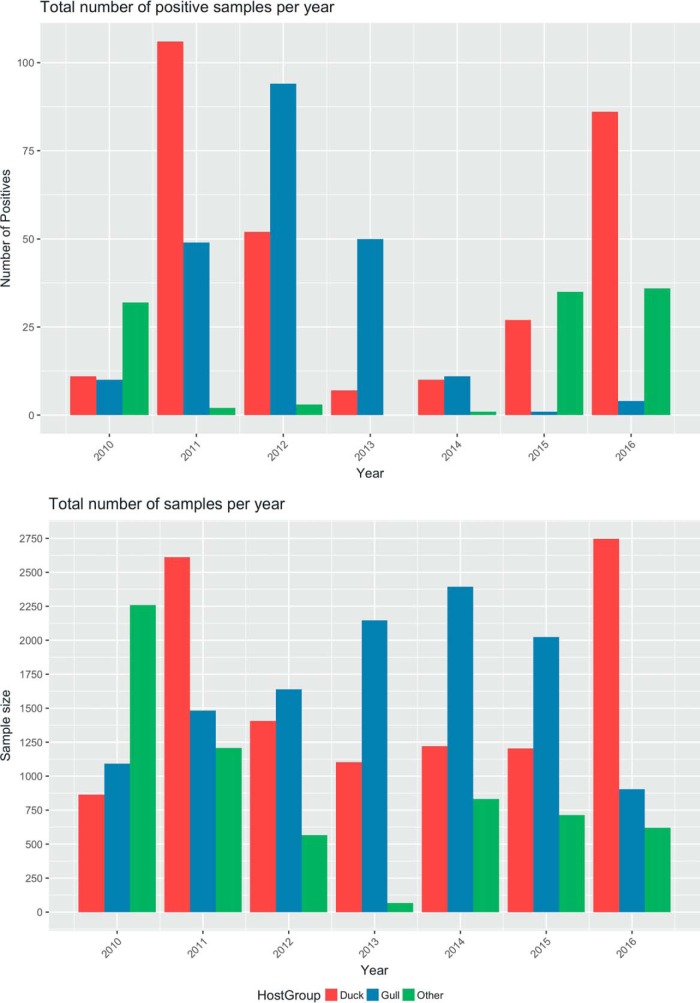
Bar chart showing the total number of positive samples (top) and the total number of samples (bottom) collected each year. The bars are colored according to the host from which samples were isolated.

**FIG 2:**
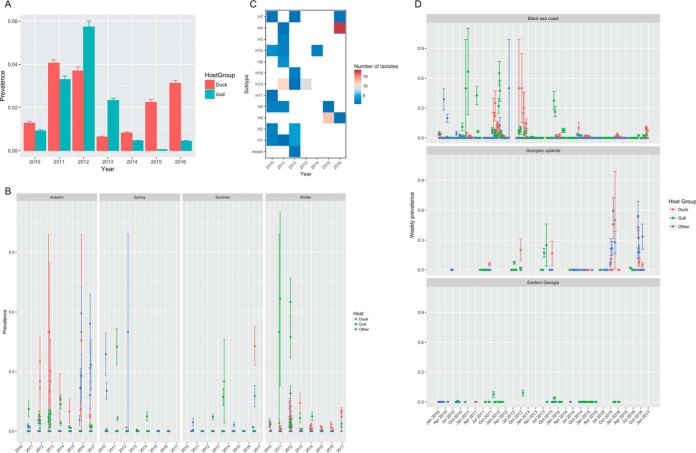
Yearly prevalence of viruses in Georgia from 2010 to 2016 overall (A), seasonally (B), by HA subtype (C), and by region (D). (A) Prevalence of virus ± standard deviation. Bars are colored according to the host from which the virus was isolated. (B and D) Prevalence of virus, with the upper and lower bounds being the 95% confidence intervals. (C) Heat map of the HA subtypes of the viruses isolated. The squares are colored according to the number of isolates of each type identified.

 Over the 6-year period between 2010 and 2016, 30,911 samples from 105 different bird species were analyzed for the presence of AIVs. Approximately 3,000 to 5,000 samples were collected every year. The total number of samples collected and the total number of positive samples for each host group each year are shown in Fig. 1. The prevalence of AIVs varied from year to year and between the two major host groups (gulls and ducks). Between 2010 and 2012, the prevalence of AIVs between gulls and ducks was comparable (Fig. 2A). The fall in prevalence in gulls from 2013 onwards could be partially explained by reproductive failure in consecutive years of two of the gull species (yellow-legged and Armenian gulls [YAG]). The data also show strong seasonality, with most positive samples being collected during the autumn migration season (Fig. 2B). When we consider the three different regions of sampling sites (Fig. 2D), we see that most of the positive gull and duck samples from 2010 to 2012 were sampled from the Black Sea coast region. After the installation of a duck trap in 2015 in the Javakheti Uplands, there was an increase in prevalence in ducks (and other birds) from 2015 onward in the uplands during the migratory season.

Twenty-four HA/NA subtypes of influenza A virus, including 12 different HA subtypes (H1, H2, H3, H4, H5, H6, H7, H9, H10, H11, H13, and H16), were isolated (Fig. 2C). The diversity of subtypes varied from year to year and was associated with the level of prevalence in duck versus gull hosts. Moreover, only a proportion of those samples that tested positive yielded virus isolates which could be typed and sequenced. Within our sampling in Georgia, the H9 and H13 subtypes were found exclusively in gulls, while H1, H5, and H7 were detected exclusively in mallards. H3, H4, H6, and H10 were found in mallards and various other ducks. Positive evidence for multiple-species infection (ducks and gulls) was found only for H2 and H11 viruses in this data set, even though globally many other subtypes are found in multiple hosts.

Between 2010 and 2012, up to seven different HA subtypes were found every year, consistent with the relatively high prevalence in both host groups in these years. The subtypes included H1, H2, H3, H4, H6, H10, H11, H13, and H16. H13 was found in the greatest proportion of sequenced samples in 2011 and 2012 and was the sole HA subtype sequenced in 2013. In 2014, again, only a single subtype was found (H10). The absence of more subtypes in these years could be explained by the comparatively low prevalence of AIVs in these years in both gulls and ducks in 2014 and especially ducks in 2013 (Fig. 2A). In 2015, the prevalence was nearly zero in gulls, but in ducks, we saw HPAI H5 type viruses detected along with an H6 virus. H4, which was previously isolated only in 2011, was the predominant type in 2016, followed by H5 and H7.

### Genetic structure of AIVs detected in Georgia from 2010 to 2016.

**FIG 3:**
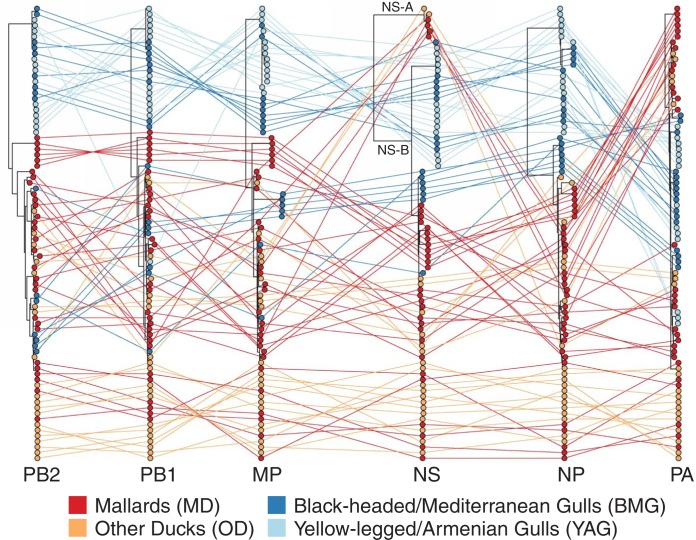
Maximum likelihood trees for all internal genes (PB2, PB1, MP, NS, NP, and PA) from equivalent strains connected across the trees. Tips and connecting lines are colored according to host type: black-headed and Mediterranean gulls (BMG), yellow-legged and Armenian gulls (YAG), mallards (MD), and other ducks (OD).

**FIG 4:**
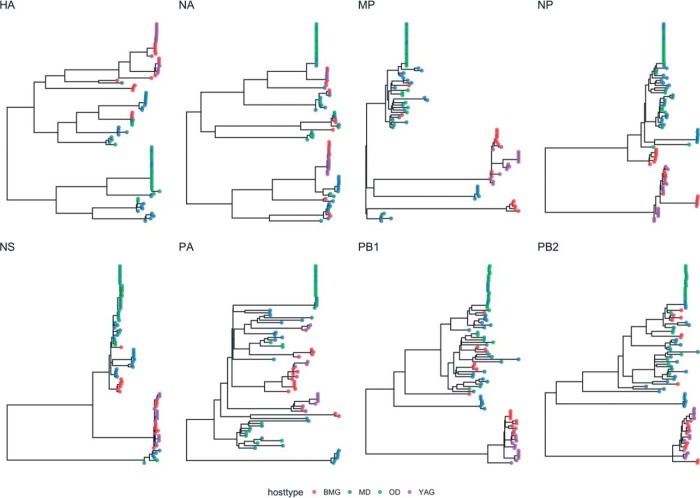
Maximum-likelihood trees for each gene segment of AIVs isolated in Georgia from 2010 to 2016. Branch supports are indicated by the approximate likelihood ratio test (aLRT) values. Tip labels are colored according to the type of bird that the strain was isolated from: black-headed and Mediterranean gulls (BMG), yellow-legged and Armenian gulls (YAG), mallards (MD), and other ducks (OD).

For all gene segments except PA, there were two major subdivisions in tree topology: one clade containing sequences predominantly from ducks and one clade entirely derived from gull sequences (Fig. 3 and 4). The internal protein-coding gene segments from certain subtypes formed subclades that were defined by the year of circulation, suggesting single-variant epidemic-like transmission within the population. This was seen in H13N8 in gulls and H4N6 and H5N8 in ducks. There were several examples of gull-derived viruses which had several internal gene segments (other than NP) located in the duck clade, and these were mostly derived from black-headed and Mediterranean gulls (BMGs). Only the PA gene phylogeny had an occurrence of a small subclade of yellow-legged and Armenian gull (YAG)-derived viruses clustered within the duck-derived viruses. For the M gene segment, there were two major clades entirely defined by host species (except for 2 BMG viruses) and an outlier subclade consisting of H2 and H9 gull lineage viruses from BMGs. For PB1, PB2, and PA, these outlier subclade viruses were found in various configurations in the tree. For NS, the tree topology divided into two alleles, as reported previously (26). However, there were only six viruses from allele A isolated from four mallards (MD), a garganey (other ducks [OD]), and a common teal (OD). Allele B split into two subclades, again defined by whether the viruses were isolated from gulls or ducks. The duck subclade included the outlier BMG viruses identified above for M. The long branch length to the gull subclade from the duck subclade in allele B would suggest that there might be host specificity in NS evolution, perhaps in response to differences between avian host innate immune responses.

### Variation in nucleotide sequence diversity.

**FIG 5:**
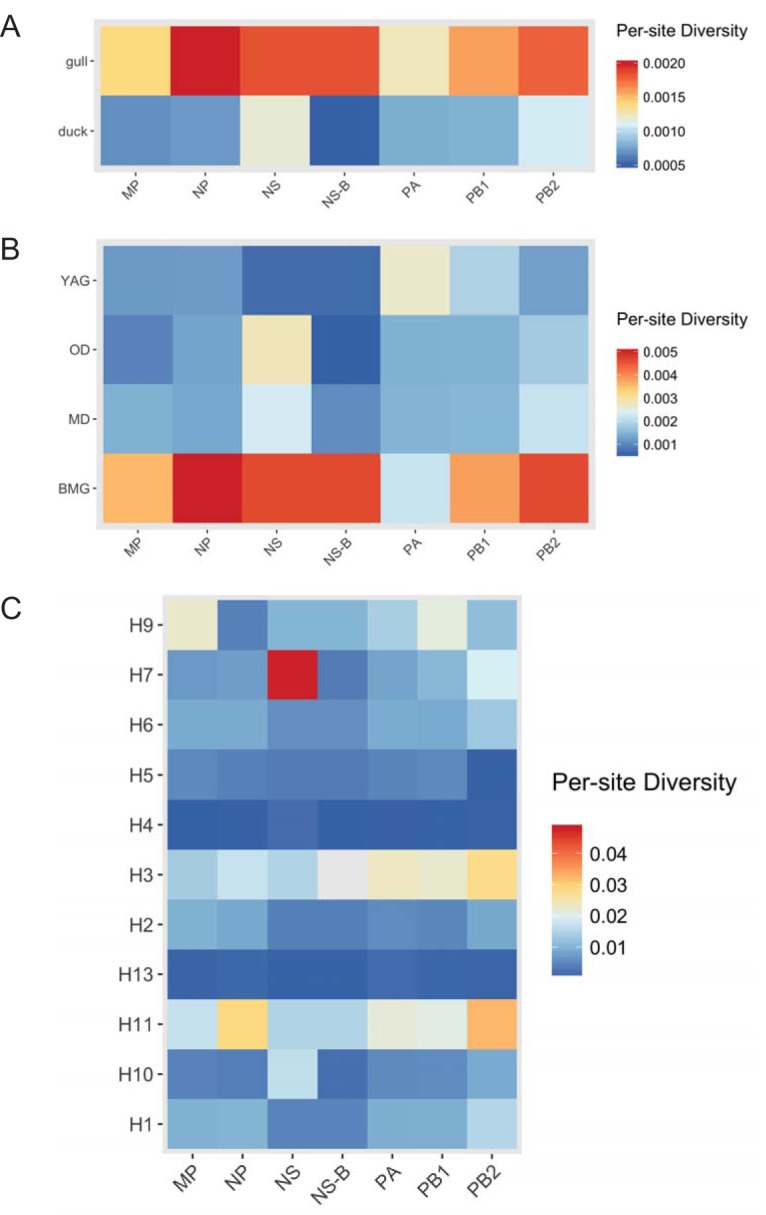
Overall per-site nucleotide sequence diversity, defined as the average number of nucleotide differences per site between two sequences in all possible pairs in the sample population normalized to the number of sequences in each population. (A) Comparison between gulls and ducks; (B) comparison between host types (black-headed and Mediterranean gulls [BMG], yellow-legged and Armenian gulls [YAG], mallards [MD], and other ducks [OD]); (C) comparison between HA types.

We used the PopGenome package in R to calculate the per-site nucleotide sequence diversity for all internal gene segments (Fig. 5A to C). The nucleotide sequence diversity of the internal gene segments in one surveillance site may be an indication of the breadth of sources from which the viruses were derived. We found greater diversity in both gulls and ducks in gene segment NS (possibly because of the presence of both the A and B alleles of this gene [NS-A and NS-B, respectively] in the data set) and PB2 (Fig. 5A). When further subdivided into host types as described in the Materials and Methods, we found that the group of black-headed and Mediterranean gulls (BMG) had the highest per-site diversity. In comparison, the mallards (MD), the yellow-legged and Armenian gulls (YAG), and other ducks (OD) had relatively lower values across all internal gene segments, even though OD comprised a variety of ducks. Only the PA gene had a greater diversity in yellow-legged and Armenian gulls than in black-headed and Mediterranean gulls (Fig. 3B). When subsets were established by HA subtype (Fig. 5C), the internal gene segments associated with H4 and H13, the most abundant types found in our data set, had the lowest diversity, possibly because several of the isolates were detected at the same time. Those less commonly isolated, such as H11, were detected in different years (2011, 2014), which may explain the high diversity of their NS, M, NP, PA, PB1, and PB2 gene segments. However, H3 subtype viruses, which also have relatively high diversity, were detected at the same time as each other (September 2011). Both the NS and NS-B data sets were used in the analysis, and as expected, the exclusion of sequences of NS-A (found exclusively in viruses from duck hosts) lowered the overall diversity within the ducks even when the values were normalized for the number of sequences found in each subset.

**FIG 6:**
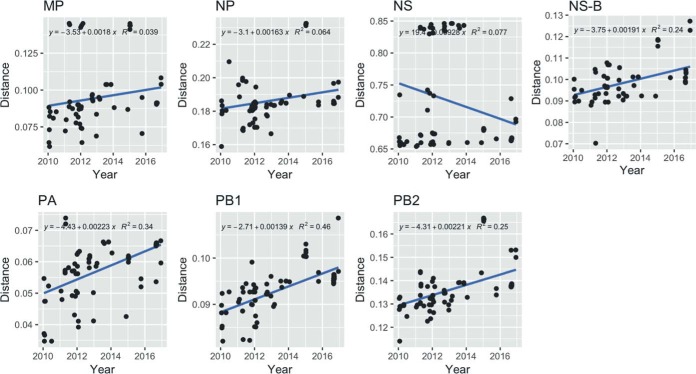
Root-to-tip regression for ML trees generated from each internal gene of viruses (MP, NP, NS [NS-A and NS-B], the NS-B allele only, PA, PB1, and PB2) isolated from Georgia from 2010 to 2016, determined using Tempest (v1.5) and plotted in R (v3.2).

**FIG 7:**
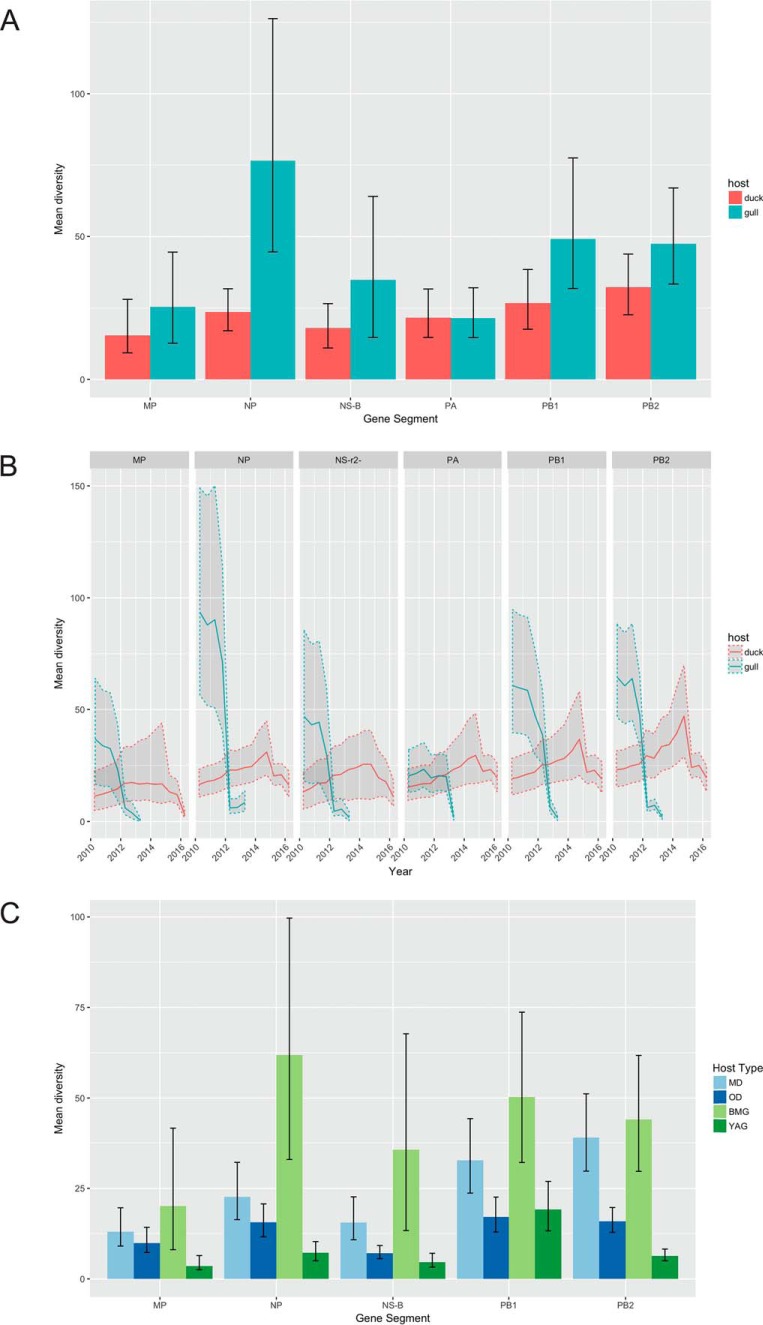
(A and B) Overall/summary (A) and overtime/skyline (B) mean diversity for each segment from gulls and ducks determined by PACT (posterior analysis of coalescent trees). Here, diversity is defined as the average time to coalescence for pairs of lineages belonging to each host. (C) Overall/summary mean diversity values for ducks, divided into mallards (MD) and other ducks (OD), and gulls, divided into black-headed and Mediterranean gulls (BMG) and yellow-legged and Armenian gulls (YAG).

We tested the root-to-tip regression for maximum likelihood (ML) trees for each of the six internal protein-coding gene segments using the Tempest (v1.5) program (27) to look for temporal signatures. All genes except the NS gene showed a positive correlation of distance with time, despite the short window of 6 years (Fig. 6). NS root-to-tip regression showed a negative slope, and it was likely confounded by the presence of two alleles, alleles A and B. Therefore, only the NS-B allele, which forms a dominant portion of the NS gene segments in the data set (75 out of 81) and shows clock likeness (Fig. 6), was used for further analysis using BEAST (v1.8.4) software. PACT analysis showed that the overall and yearly host-related diversity measures (Fig. 7A and B) showed similar trends, as seen in Fig. 5.

### Correlation of traits with phylogeny.

**FIG 8:**
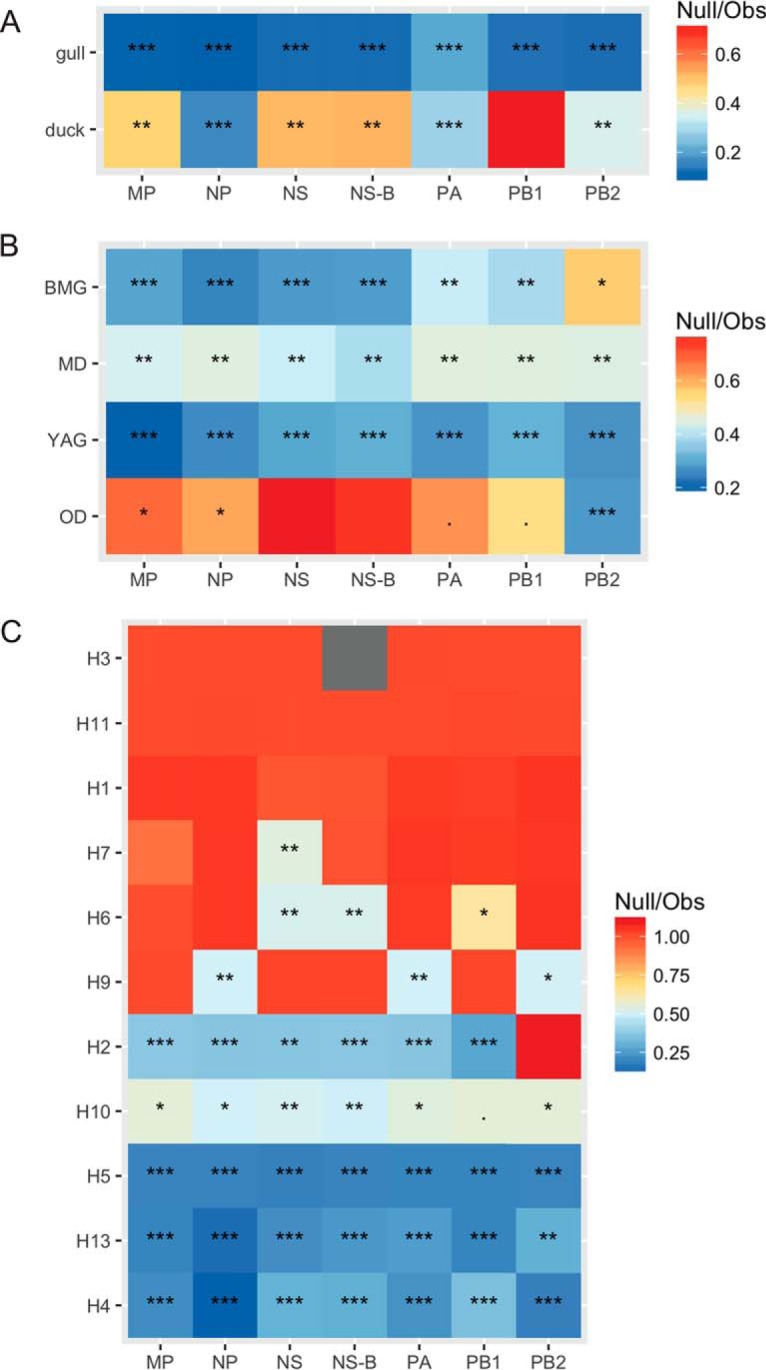
Summaries of expected/observed (Obs) ratios from Bayesian tip-association significance testing (BaTS) for all internal genes. Higher values indicate less phylogenetic clustering by trait and, hence, higher rates of mixed ancestry. Comparisons between gulls and ducks (A), host types (BMG, black-headed and Mediterranean gulls; YAG, yellow-legged and Armenian gulls; MD, mallards, OD, other ducks) (B), and HA types (C) are shown. Asterisks indicate *P* values (***, *P* < 0.001; **, *P* < 0.01; *, *P* < 0.05; no asterisk, *P* > 0.05).

We tested the null hypothesis that there is no association between phylogenetic ancestry and traits (host group, host type, and HA subtype) using Bayesian tip-association significance testing (BaTS). The ratios of clustering by each trait on the gene segment trees that is expected by chance alone (null mean) to the association that is observed in the data (observed mean) are presented in Fig. 8A to C. The higher that the value of null/observed is, the lower is the support for phylogenetic clustering of the given trait. Therefore, a higher value indicates a different ancestry. Hence, when we consider the HA subtype trait to be the lineage, it provides a measure of reassortment, as described previously (28). Again, the NS-B data set was considered along with the complete NS data set, but no significant differences in trends were found. Figure 8A shows that gull viruses were more likely to cluster together in a phylogenetic tree than duck viruses in general. When the viruses of gulls and ducks were further subdivided, Fig. 8B shows that OD viruses were less likely to cluster together in the tree, which is expected, given that we grouped together several duck species under this category. Among the rest, again, it was the duck species (MD) that exhibited dynamic phylogenetic placement compared to both the gull types. The only exception was with the PB2 gene segment, for which the BMG showed a lower level of phylogenetic clustering by species, indicating putative reassortment events. When we considered the HA subtype (lineage) of the viruses, we found that H4 and H13, which showed the lowest nucleotide sequence diversity, also showed very low levels of reassortment, as did H5. There was not enough statistical power to interpret events in H1, H3, H6, H7, H9, or H11 viruses. Where statistically significant values were found, lower levels of clustering were observed.

### Directionality of viral gene segment transfer.

**FIG 9:**
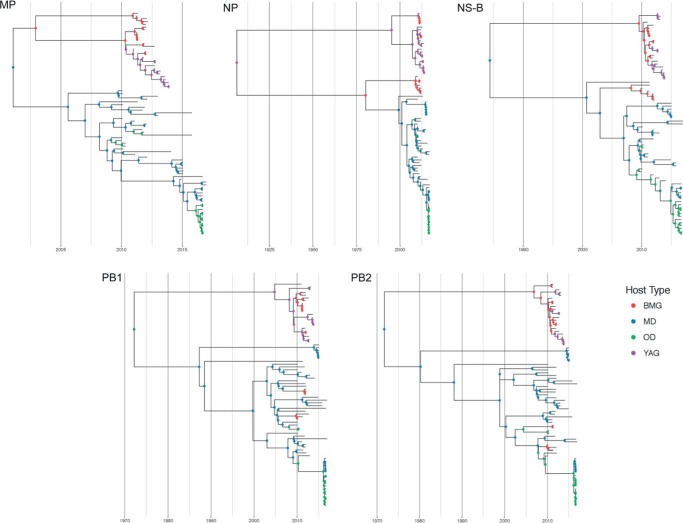
Maximum clade credibility (MCC) trees for five of six internal gene segments of AIVs isolated in Georgia from 2010 to 2016. Node icons are colored according to the host type state inferred by BEAST (v1.8.4) software. BMG, black-headed and Mediterranean gulls (red); YAG, yellow-legged and Armenian gulls (purple); MD, mallards (blue); OD, other ducks (green).

**FIG 10:**
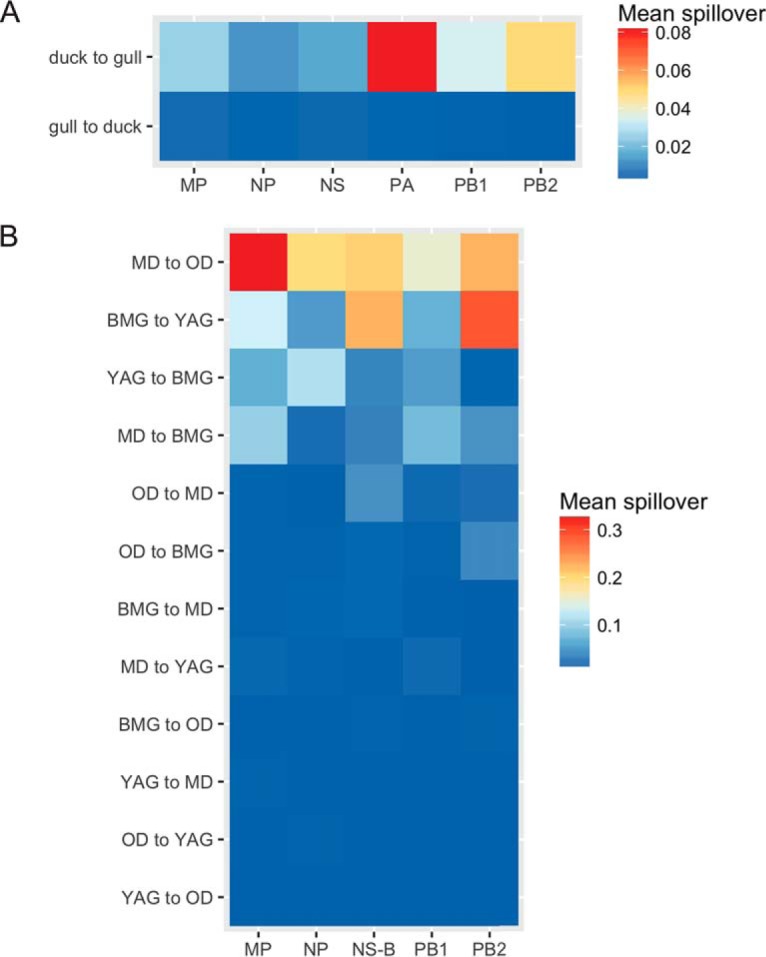
Summary of mean migration events between hosts in the direction from duck to gull and gull to duck (A) and between different host types (BMG, black-headed and Mediterranean gulls; YAG, yellow-legged and Armenian gulls; MD, mallards; OD, other ducks) (B) derived from the genealogy.

Figure 9 shows ancestral reconstructions of the host state along time-scaled phylogenies for five of six internal gene segments. The results are summarized in Fig. 10A, showing the mean number of host jump events from duck to gull and vice versa. For all gene segments, most of the host spillover events are in the direction from ducks to gulls. In Fig. 10B we see that at a finer level, most of the host jump events happened within the duck (mallards [MD] to other ducks [OD]) and gull (black-headed and Mediterranean Gulls [BMG] to yellow-legged and Armenian gulls [YAG] and vice versa) species. In transmissions from ducks to gulls, it is largely noticeable only from MD to BMG. This likely explains the higher levels of nucleotide sequences diversity and reassortment rates in the BMG viruses than in the YAG viruses seen above.

### Geographical context for GE NS, M, NP, PA, PB1, and PB2 segments.

**FIG 11:**
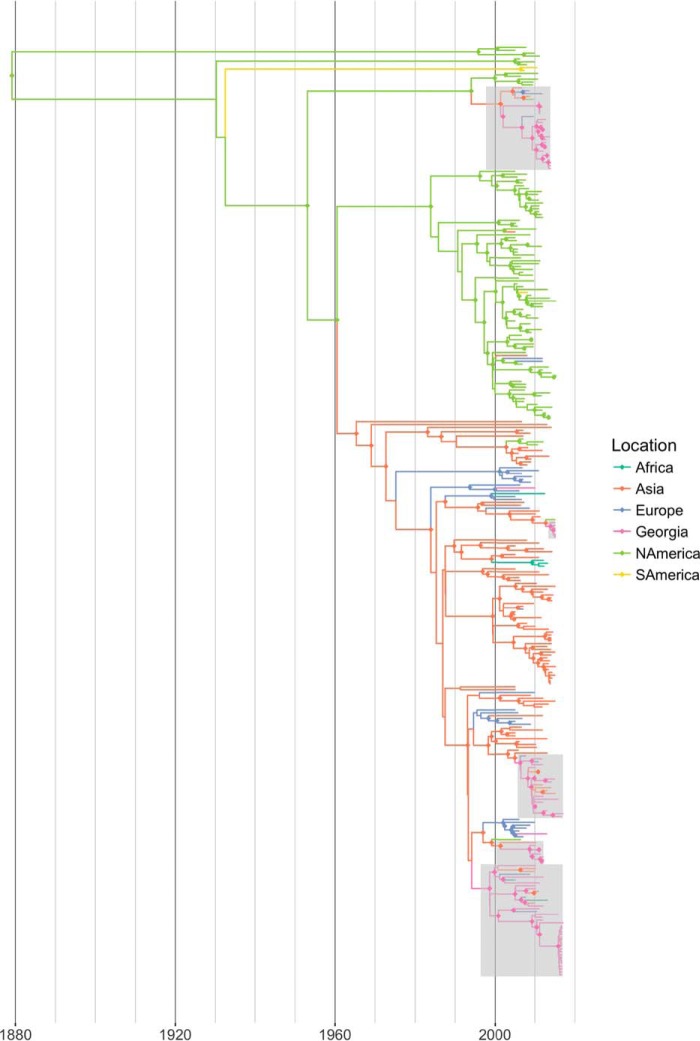
BEAST maximum clade credibility (MCC) trees from viral NP gene sequences isolated worldwide from avian hosts between 2005 and 2016. Branches are colored according to the location observed at the tips and estimated at internal nodes by ancestral reconstruction of the discrete trait. Nodes with a posterior probability of >0.85 are annotated with a diamond icon in the same color as the branch. NAmerica, North America; SAmerica, South America.

**FIG 12:**
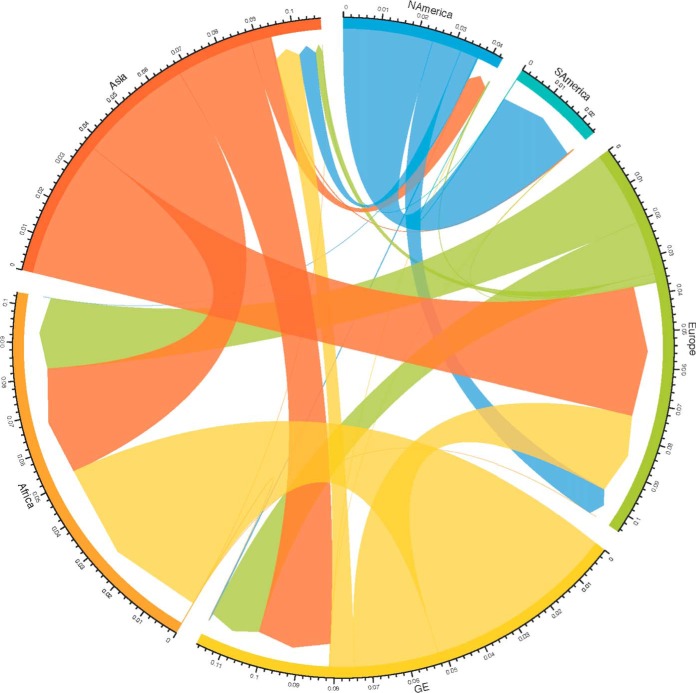
The circularized graph shows the overall rates of migration, defined as the rate at which labels (locations) change over the course of the genealogy, between Georgia and other locations. Arrowheads indicate the direction of migration; rates are measured as the number of migration events per lineage per year (indicated by the width of the arrow). GE, Georgia.

To determine the origin and destination of the internal protein-coding gene segments found in viruses isolated in Georgia, we analyzed our sequence data set together with avian influenza virus sequences from a broader time frame (2005 to 2016) and regional sampling. Figure 11 shows the genealogy for the NP gene for whose tips we knew the location of sampling and whose internal nodes were estimated using discrete-state ancestral reconstruction in BEAST software. The clades in which Georgian sequences occurred are highlighted. Figure 12 summarizes the genealogy in a circularized graph in which the arrowheads indicate the direction of transfer and the width of the arrows indicates the rate of transfer to different locations. The analyses reveal that viruses from the Atlantic and Afro-Eurasian locations form largely separate clades, which is consistent with the findings of previous studies (29, 30). However, we did find instances of transmission across this divide, most notably, to and from Asia and Europe. Many NP genes from Georgia clustered with other Georgian NP genes, in some cases forming the terminal branches spanning years indicating restriction to local spread. However, our data set contains the latest Georgian sequences, and sequences from this time frame were not available from the rest of Eurasia. Hence, we can expect to have missed identifying onward transmission. From the transmission that we did identify, it appears that there is considerable migration into Africa and Europe and to a lesser extent into southern/eastern Asia. Most of the sequences transmitted into Georgia came from Asia and Europe, along with a single identified instance of direct transfer from North America.

## DISCUSSION

Wild birds have been shown to harbor avian influenza viruses with a substantial genetic diversity. This study showed that the diversity not only varied by year but also was associated with the level of overall prevalence in different wild bird host species, perhaps influencing the observed rates and diversity if prevalences were low. We observed ecological fluctuations during the study period which might have influenced the results. In 2015, there was a nearly complete reproductive failure in the breeding colony of Armenian gulls, which might have resulted in few susceptible juveniles and therefore altered influenza prevalence. In 2013, the nest sites on the Chorokhi River Delta were flooded consecutively, again perhaps influencing disease dynamics. While the installation of the duck trap in the Javakheti Uplands improved the longitudinal window of duck sampling to include both overwintering and migratory populations, this initiative might have introduced prevalence and subtype biases in the data by sampling a previously unsampled subpopulation. However, even allowing for these biases, the results from this study show that there is little evidence that one species group maintains all influenza A virus diversity, there appears to be relative host restriction in many subtypes (except for H2 and H11 viruses), and there are differences in prevalence dynamics depending on the host. Therefore, the influenza A viruses in one host species are not representative of the influenza A virus prevalence, dynamics, and diversity across the wild bird reservoir. Within both ducks and gulls, however, peak prevalence was consistently observed in hatch-year birds and with a more restricted subtype diversity, suggesting that there was an initial influenza A virus epidemic wave as naive birds aggregate in their first year. Subsequently, in the overwintering period, a wider subtype diversity was observed in both host groups, and adults were more frequently infected. This suggests that disease dynamics are complex and influenced by multiple host factors, including age and annual life cycle stage.

It has previously been observed that some subtypes are routinely and nearly exclusively isolated from certain host families/genera, with the most notable example being H13 and H16 viruses from gulls. However, mixed infections are relatively common but might be masked if subtype characterization requires virus isolation, therefore putting the clinical specimen through a culture bottleneck. Advances in sequencing directly from clinical material would more accurately remove possible culture selection bias and establish the prevalence, subtype diversity, and genetic diversity within wild birds.

In general, for all gene segments except PA, we identified strong patterns of clade topology defined by host. This suggests that there is segregated gene flow through these host populations with little interhost reassortment. Additionally, within our study period there were large-scale perturbations in ecology which might also have influenced our prevalence and subtype diversity estimates. For example, in 2014 and 2015 there was widespread reproductive failure in two gull host species due to nest flooding (yellow-legged gulls) and few adults returning to the colony (Armenian gulls) and, therefore, few juveniles from which to detect the annual epidemic wave. The occurrence and significance of such ecological fluctuations on disease dynamics are unclear. We also increased the ability to sample migrant ducks in late summer and early autumn from August 2015 by constructing a duck trap in the newly created national park. Again, this addition to the sampling strategy likely increased the detection of influenza viruses in these anseriform hosts, as they were previously undersampled.

We tested whether certain hosts maintained higher levels of nucleotide sequence diversity in the non-immune-related internal genes. PB2 and NS were the most genetically diverse in both gulls and ducks. Within the host groups, black-headed and Mediterranean gull-derived viruses showed the highest per-site diversity and yellow-legged and Armenian gull-derived viruses showed a lower diversity, likely because some of the viruses of the former were associated with reassortants probably derived from ducks (or another unsampled host group). Despite high rates of reassortment and spillover between duck subgroups, mallards (MD) and other ducks (OD), the absence of any gull-derived viruses in these ducks keeps their diversity levels lower than those in gulls/BMG.

Where gene flow does occur between host groups, for all gene segments, host spillover events were in the direction of ducks to gulls and from other ducks to black-headed and Mediterranean gulls, likely explaining the higher levels of nucleotide sequence diversity in these gulls that were observed, as described above. Where HA and NA gene segments were acquired by gulls from ducks, there was a prerequisite for a gull clade internal gene cassette, suggesting a host-restrictive effect for onward maintenance within the gull population (13, 31). Interestingly, black-headed and Mediterranean gulls occurred on the study site only in the overwintering period, when there were also high densities of overwintering ducks from other geographic areas. Although there is a duck-gull interface on the breeding grounds in summer, the duck densities are very much lower, perhaps suggesting that there is a threshold level of bird density that allows gene flow among hosts.

If we look at diversity by HA subtype, H4 and H13 were the least diverse, showed the lowest rates of reassortment, and were also associated with hatch-year bird infections, suggesting a clonal expansion and epidemic gene flow through these birds. The HPAI H5 viruses responsible for the 2014-2015 epizootic also showed no reassortment, unlike the HPAI H5 viruses from the 2016-2017 epizootic, perhaps indicating that the first wave of clade 2.3.4.4 viruses diffused through the wild bird population similarly to a naive infection, and subsequent epizootics have resulted in altered pathogen evolution strategies to maintain gene flow, similar to those previously observed in North America, when considering the effect of latitude on gene flow (7).

When we examined the internal gene segments of the Georgian AIVs in a broader geographic context, we found significant gene flow to Georgia from Europe and the rest of Asia and from Georgia to Europe and the rest of Asia, although data for Africa are very limited. Crossover into the Atlantic flyway appears to be mediated largely by gulls, with some exceptions, notably, the H5N1 NP gene, which was transmitted between ducks.

From this study, the diffusion of avian influenza viruses within a multihost ecosystem is heterogeneous. One host group therefore cannot be used as a surrogate for others. It is likely that virus evolution in these natural ecosystems is a complex mix of host-pathogen interface and ecological factors. Understanding such drivers is key to investigating these emerging pathogens, interpreting the data from different sites around the world, and ultimately, informing the risk of incursion of emerging variants from one geographic region to another.

## MATERIALS AND METHODS

### Surveillance.

Active surveillance for influenza A viruses was carried out from 2010 to 2016 in the Republic of Georgia as described previously (15). The study area and sample collection methods remained predominantly the same as those described previously (15). In this analysis, the study area was divided into three groups on the basis of the bird annual life cycle and geography: the wetlands in Ajara, Guria, and Samegrelo constitute the Black Sea coast region; Samtskhe-Javakheti forms the Georgian uplands sampling area; and finally, Tbilisi and Kakheti are grouped as eastern Georgia. Sampling was targeted toward the Anatidae (ducks), Charadriformes (gulls), and other birds commonly found in the wetland ecosystems. Details of the host species considered can be found in reference 15. We used several methods to catch birds depending on the species and location, including mist nets, spring traps, and manual capture using hand-held nets; lamping; and sampling hunted birds. We took paired oropharyngeal and cloacal swab specimens, serum specimens, and in some cases feather samples from all live-caught birds.

To sample live-caught or hunted birds, a sterile plain cotton swab was inserted into the trachea or oropharynx (in smaller bird species) or approximately 5 mm into the cloaca of the bird and then gently turned to moisten the swab. All swabs were then inserted into viral transport storage medium (Hanks balanced salt solution containing 10% glycerol, 200 U/ml penicillin, 200 mg/ml streptomycin, 100 U/ml polymyxin B sulfate, and 250 mg/ml gentamicin), and the shaft of the swab was broken just above the cotton tip. The swabs were stored at −70°C for no more than 6 h after collection and were chilled at 1 to 4°C on ice or in a portable refrigerator in the interim. Surveillance was carried out throughout the year, but there was seasonal fluctuation in bird density. In addition to previously described methods, we built a duck trap in the Javakheti Uplands close to the gull colony sampling site in 2015.

### Data set and genomic sequencing.

Over a period of 6 years, 30,911 samples from 105 different bird species were analyzed for the presence of AIVs. Isolates were obtained by standard approaches (32), and where possible, the isolates were subtyped and a sequence was generated from extracted RNA as described below.

For virus samples collected from 2010 to 2012, codon-complete genomes of IAVs were sequenced as part of the Influenza Genome Project (http://gcid.jcvi.org/projects/gsc/influenza/index.php), an initiative by the National Institute of Allergy and Infectious Diseases (NIAID). IAV viral RNA (vRNA) was isolated from the samples/specimens, and the entire genome was amplified from 3 μl of RNA template using a multisegment reverse transcription-PCR (RT-PCR) strategy (M-RTPCR) (33, 34). The amplicons were sequenced using an Ion Torrent PGM (Thermo Fisher Scientific, Waltham, MA, USA) and/or an Illumina MiSeq (v2; Illumina, Inc., San Diego, CA, USA) instrument. When sequencing data from both platforms were available, the data were merged and assembled together; the resulting consensus sequences were supported by reads from both technologies. Sequence data for Georgia were downloaded from the NIAID Influenza Research Database (IRD) (35) through the website at http://www.fludb.org on 5 November 2016. To this data set we added sequence data for isolates from 2013 and 2016, which were sequenced at either the Erasmus MC, the Animal and Plant Health Agency (APHA), or the Icahn School of Medicine at Mount Sinai (ISMMS). At Erasmus MC, sequencing was performed as described previously by Munster et al. (36), with modifications. Primer sequences are available upon request.

At APHA, viral RNA was extracted using a QIAquick viral RNA extraction kit (Qiagen, UK) without the addition of carrier. Double-stranded cDNA (cDNA synthesis system; Roche, UK) was generated from RNA according to the manufacturer's instructions. This was quantified using the fluorescent PicoGreen reagent, and 1 ng was used as a template for the preparation of the sequencing library (NexteraXT; Illumina, Cambridge, UK). Sequencing libraries were run on a MiSeq instrument (Illumina, Cambridge, UK) with 2 × 7-base paired end reads. Handling of the data from the raw sequence reads and extraction of consensus sequences were performed at APHA.

At the Icahn School of Medicine at Mount Sinai, RNA was extracted using a QIAamp viral RNA minikit (catalog number 52904; Qiagen, UK). M-RTPCR amplification was performed with a SuperScript III high-fidelity RT-PCR kit (catalog number 12574-023; Invitrogen) according to the manufacturer's instructions using the Opti1 primer set, consisting of primers Opti1-F1 (5′-GTTACGCGCCAGCAAAAGCAGG), Opti1-F2 (5′-GTTACGCGCCAGC**G**AAAGCAGG), and Opti1-R1 (5′-GTTACGCGCCAGTAGAAACAAGG). DNA amplicons were purified using an Agencourt AMPure XP 5-ml kit (catalog number A63880; Beckman Coulter). At the Icahn School of Medicine at Mount Sinai, sequencing libraries were prepared and sequencing was performed on a MiSeq instrument (Illumina, Cambridge, UK) with 2 × 150-base paired end reads. Handling of the data for the raw sequence reads and extraction of consensus sequences were performed at ISMMS as described previously (37).

### Genetic analyses. (i) Sequence alignment preparation.

Whole-genome sequences from 81 Georgian strains isolated between 2010 and 2016 were used in this analysis. We aligned sequences from each gene segment separately using the MAFFT (v7.305b) program (38) and trimmed to starting ATG and stop codons in the AliView (v1.18) alignment viewer and editor. Hemagglutinin (HA) sequences were further trimmed to exclude the initial signal sequence (39, 40). Sequences were then aligned using the muscle-codon option with the default settings in MEGA7 software (41).

The NS gene has two alleles, alleles A and B, with a significant difference in sequence composition, which could skew the analyses of sequence diversity. The NS gene sequences were therefore considered both as a complete data set (NS) and as the subdivided NS-A and NS-B data sets, where required. As only 6 out of 81 sequenced strains had the NS-A allele, only the NS and NS-B data sets were used in the analyses.

We then subdivided the complete data sets of each gene according to viral traits, namely, (i) host group (gull and duck); (ii) host type, consisting of black-headed gulls (Chroicocephalus ridibundus) and Mediterranean gulls (Ichthyaetus melanocephalus) (BMG), yellow-legged gulls (Larus michahellis) and Armenian gulls (Larus armenicus) (YAG), and mallards (Anas platyrhynchos) (MD) and other ducks (OD; which includes the common teal [Anas crecca], domestic duck [Anas platyrhynchos domesticus], garganey [Anas querquedula], northern shoveler [Anas clypeata], common coot [Fulica atra], and tufted duck [Aythya fuligula]); and (iii) HA subtype. The data set was reduced to include subtypes H1, H2, H3, H4, H5, H6, H7, H9, H10, H11, and H13, for each of which greater than three sequences were available for statistical analyses.

### (ii) Visualization of phylogenetic incongruence.

We inferred maximum likelihood (ML) phylogenetic trees for each gene segment using IQ-TREE (v1.5.5) (42) and ModelFinder (43) software and obtained branch supports with a Shimodaira-Hasegawa (SH)-like approximate likelihood ratio test (aLRT) and standard nonparametric bootstrap analysis. All trees were rooted using the best-fitting-root function in the Tempest (v1.5) program (27) and visualized in FigTree (v1.4.2) software with increasing node order. To visualize incongruence, we traced the phylogenetic position of each sequence, colored according to host, across unrooted ML trees for all internal gene segments. Figures were generated by modifying scripts from a similar analysis (44).

### (iii) Quantification of nucleotide sequence diversity.

Complete alignments of each internal gene, as well as alignment subsets by host group, host type, and HA subtype, were used in the PopGenome package in R (v3.2) (45) to estimate nucleotide sequence diversity. Per-site diversity was calculated by dividing the nucleotide sequence diversity output by the number of sites present in each alignment. As each subset contained different numbers of sequences, this value was normalized by dividing by the number of sequences in each respective data set. Heat maps from these data were plotted in R (v3.2).

### (iv) Correlating traits with phylogeny (BaTS).

 The null hypothesis of no association between phylogenetic ancestry and traits (host group, host type, and HA subtype) was tested using Bayesian tip-association significance testing (BaTS), beta build 2 (46), for all internal gene segments. Bayesian posterior sets of trees were inferred using MrBayes (v3.2.6) software (47) using the same segment-wise alignments generated for ML tree estimation. The ratio of clustering by each trait on the gene segment trees that is expected by chance alone (null mean) to the association that is observed in the data (observed mean) was calculated. These expected/observed ratios were summarized in a heat map in which the *y* axis was ordered by the amount of reassortment observed. Data manipulation and figure preparation were done in R (v3.2).

### (v) Quantification of diversity and between-host transmission.

The alignments generated for the ML trees were also used in Bayesian phylodynamic analyses using BEAST (v1.8.4) software (48). We employed a strict molecular clock, a coalescent constant tree prior, and the SRD06 site model with two partitions for codon positions (1st and 2nd positions, 3rd position), with base frequencies being unlinked across all codon positions. The Monte Carlo Markov chain (MCMC) was run twice for 100 million iterations with subsampling every 10,000 iterations. All parameters reached convergence, as assessed visually using the Tracer (v1.6.0) program. The LogCombiner (v1.8.4) program was used to remove the initial 10% of the chain as burn-in and to merge the log and trees files output from the two MCMC runs. Maximum clade credibility (MCC) trees were summarized using the TreeAnnotator (v1.8.4) program. After removal of the burn-in, the trees were analyzed using PACT (posterior analysis of coalescent trees; https://github.com/trvrb/PACT.git) to determine measures of diversity and the migration rates between hosts over time.

### (vi) Geographical context for Georgian origin internal protein-coding gene segments.

Internal gene sequences from avian hosts sampled across the world between 2005 and 2017 were obtained from gisaid.org (downloaded November 2017). The sequences (each segment separately) were divided into regions, namely, Asia (including Oceania), Europe, Africa, North America, and South America. The program cd-hit-est (49, 50) was used to down-sample each regional data set to a 0.9 similarity cutoff level. These down-sampled sequences were then merged with the Georgian data set. Discrete trait ancestral reconstruction with symmetric and asymmetric models was implemented in BEAST (v1.8.4) software (48), together with marginal likelihood estimation using path-sampling/stepping-stone analysis. The symmetric model was chosen over the asymmetric model (log Bayes factor = 14). The MCMC was run twice for 100 million iterations with subsampling every 10,000 iterations. All parameters reached convergence, as assessed visually using the Tracer (v1.6.0). LogCombiner (v1.8.4) was used to remove the initial 10% of the chain as burn-in and to merge the log and trees files output from the two MCMC runs. Maximum clade credibility (MCC) trees were summarized using TreeAnnotator (v1.8.4). PACT was used to extract overall migration rates between trait locations.
